# Veterinary Medicine

**Published:** 1838-01

**Authors:** 


### VETERINARY MEDICINE.

*Observations on Rabies in Animals.* By Dr. Wagner, Medico-Forensic Censor of the Schieben District.

[The following observations are interesting and valuable, on account of their authenticity. We particularly recommend them to the notice of gentlemen practising in the country, who may have opportunities of witnessing similar cases. We are firmly convinced that medicine is destined to derive no slight advantages from observations made on the diseases of animals; and, on this account, as well as others, we are gratified in observing the gradual, but still too slow, improvement in the pathological knowledge of our veterinary surgeons.]

1. *In the Dog.* A dread of water is not a symptom invariably attending rabies. I have met with two instances of men being bitten by dogs, and dying from rabies, when the dogs, nevertheless, ate and drank shortly after having made the attack. One of these was seen to swim through the river Elster on the ensuing day. Another, in the same state, displayed great activity in the water for several hours, in search of wild fowl, until, on a sudden, it ceased to recognize its master, appeared to have lost the senses of sight and hearing, rushed out of the water, and scampered away, snapping on the road at every object within its reach.

Though rabid dogs are frequently in the habit of gnawing at wood, straw, or hides, snapping round about in the air, &c., such signs are equally fallacious with the foregoing one, healthy dogs being sometimes observed to do the same.

An appearance of dejection and shyness, a hanging of the head and tail, running at the eyes, or the fierce flashing eye without this, foaming at the mouth, refusal of food and drink, are all suspicious symptoms, and demand caution; but, as they are attendant upon other canine diseases likewise, they are not conclusive. In fact, experience has furnished me with no evidence *decisive* of the existence of rabies, except that of a dog running away and recklessly attacking both men and animals, especially those of its own species. No other dog, even the most vicious, when accidentally separated from its master, will attack any save animals of its own kind, unless in self-defence. In a rabid state, some dogs will even leap over high fences, in order to reach dogs or cats which they discover to be on the other side; a proof that scent, sight, and hearing are still unimpaired. Others will sneak along a wall, or run forward in a straight line, attempting to bite whatever they thus meet with, but never diverging from their headlong course, unless to pursue other dogs. I have observed some rabid dogs which never moved from one spot, merely gnawing at everything within reach, as they lay, snapping at vacancy, and ultimately refusing food and drink, or, if they attempted to partake of either, appearing incapable of deglutition. In these cases, palsy of the loins and hind extremities appears to have taken place at the outset, and to have deprived the animals of the power of locomotion. Drooping of the tail and head, and foaming at the mouth, are, of all signs, the least to be relied on: they belong only to the last stage of the disorder, when the animal has almost ceased to live. On the other hand, it is a well-known fact that some dogs, in a state of health, constantly foam at the mouth, and that all look dejected and hang their tails when they have wandered astray. The same observation applies to the absence of barking, or to the hoarse bark: many dogs bark but rarely, whilst with others the bark is perpetually hoarse. On the contrary, many rabid dogs continue to growl or raise a howling bark, (so that a single short bark is succeeded by a shrill lengthened howl,) similar to that made by healthy dogs at certain sights or sounds, from hunger, thirst, &c. Even in the appearance of the eye in rabies, the unprejudiced observer will not discover any essential change.

The moment a dog evinces any traces of illness, it is no longer to be trusted; and it would be well to lock it up, or fasten it to a stout chain. But, when it begins to gnaw wood, to show a dull eye, to snap at animals with which it had become familiarized, to bark hoarsely; when it attempts to run away or to break its chain, eats and drinks with a snapping gesture, at intervals appears lively, and then again sneaks sulkily to its kennel; when it disregards its masters' call, and, contrary to its former habits, growls and snarls at well-known persons, the animal ought to be despatched; for there can no longer remain a doubt of its being rabid. If a dog runs away, and returns home on the second or third day, with any unhealthy symptoms, its rabidness is equally certain, more particularly if it then ceases to know its master.

The rabid dog is often very sudden in its motions, darting at its victim with the quickness of lightning. In the first stages it bites slily, and rather pinches than wounds; but, at a later period, the bite is so terrible, that force is sometimes necessary to disengage the animal. I witnessed two cases in which the dogs could not be made to relax their hold until their spine had been divided by a blow with a hatchet.

2. *In Oxen.* In horned cattle, amongst which I have met with the most numerous instances of rabies, this disorder evidently occurs under two distinct forms. In solitary cases, where we are not previously aware of the animal having been bitten, we should scarcely be led to a suspicion of rabies. The commencement and progress are as follows:—With a healthy exterior and clear eye, the animal loses its appetite, eats and drinks by fits only, without however refusing choice morsels that may be offered to it; at times it appears as if suddenly stupified, recedes from the manger, and forgets to chew, then again chews on, listens with liveliness to every noise that is made, seems to notice every object, continues to obey its keeper, and sometimes remains capable of working: borborygmi are, however, already to be heard, and there is sometimes observable a slight straining or disposition to tenesmus. In the cow, the secretion of milk decreases, but by no means ceases at the very commencement. In the open air, the animal ceases to graze, appears lost, strays from the rest of the herd; although when, by itself, it generally allows itself to be led quietly back to the stable.

After the lapse of from one to three days, all the symptoms increase; neither hunger nor thirst is experienced, although no dread of water is apparent, and the animal still ruminates at intervals. The eyes have commonly a healthy look, but flash at times, without seeming inflamed. The creature lows but seldom; but, when it does, several times consecutively, and either with a hoarse tone, or, on the contrary, in so clear and powerful a key, that I have heard it distinctly at a distance of more than a mile. The mouth and tongue have still nothing abnormal about them. The borborygmi increase. At the same time the animals are observed to lick some particular part of their bodies, especially one of the feet, (probably the spot where they were bitten,) until that part becomes excoriated and bleeds. Palsy of the loins now ensues; the animal remains lying, and, if forced to rise, which it does with difficulty, totters on its hind legs; an increased straining is remarked at the anus, and this is sometimes followed by hard but ultimately by thin evacuations; it moves its head alternately from side to side, and appears anxious to lick at cloth or fur, &c., which it by degrees gets between its teeth, and tears. To this the suffering animal appears not to be urged by any vicious propensity. At this period, in the cow, the secretion of milk is at an end.

The above symptoms continue on the increase until the sixth or seventh day. At length the hind extremities become so paralysed as to render standing on the feet impossible; the constant straining at the rectum still forces out fæces, and the animal refuses all, or almost all, further sustenance; even bread that is forced with the hand into the creature's throat is not swallowed. A dread of water is never observed, even though the latter be refused. Naturally thin and rough-haired cattle become still thinner, and their hair rougher; but such as were originally in good condition and smooth haired show little alteration in either respect. Some time between the sixth and the ninth day, the animals sink on one side, (mostly, I have observed, on the left,) with the head commonly stretched backwards; the eye still remaining bright, lively, and uninflamed. From henceforth the trunk continues motionless, whilst the legs undergo a constant but languid movement to and fro, until the animal has ceased to exist. In the form above described, the animals may be approached with little or no danger. Not so in the second form; of which, however, scarcely one case is observed in six.

This form commences like the other. In the stable, the animals recede still more from the manger, and will even break the rope with which they are fastened, if it is not very strong. Their bellowing, without being frequent, is prolonged, its tone clear, thrilling, and unmodulated. They scratch the ground with their fore hoofs, often with such violence that the dung is projected to the roof of the stable; whilst with their hind legs they kick violently at any one who approaches. At this stage the paroxysms are periodical, and little is to be observed during the intervals, except a reluctance to feed or drink. About the fourth day, if they are naturally strong, they will snap every kind of fastening in twain during the paroxysm, and then attack and gore all who approach them. They rage about in the stable, gnawing the cribs and other objects to pieces, until palsy of the joints supervenes, when they sink down, and ultimately fall on one side. In this position they still manage, spasmodically as it were, to shove themselves about the stable by means of their hind legs. Last of all, they lie for hours on the side, as if dead, when the bright shining eye, and occasionally a convulsive movement, alone indicate a remnant of life. On the seventh, eighth, or at most the ninth, day, they die.

On dissection, nothing abnormal is to be discovered beyond a gall-bladder filled to excess with dirty, yellowish-green coloured bile in a state of complete fermentation. From the great distention of the bladder, the fermentatory process is easily distinguished from without.

3. *In Horses.* With horses the symptoms of rabies are as follows: in the team they display unwonted activity and emulation. Here, however, as well as in the stable, they assail their fellows with bites and kicks. They generally refuse both provender and water, nod or jerk the head frequently, (as horses are in the habit of doing, when they are in spirits), and have a fiery eye. On the second, or at most on the third day, these symptoms increase to such a degree that no creature can approach them without being bit and kicked in the most frightful manner, and to offer them either food or water appears only to augment their rage. Yet moments of quietness intervene, provided the stable be kept dark and nothing around them stir. On the other hand, such is their fury when disturbed, that on a plank being taken out of the stable door in order to destroy them with safety, they will rush to the spot, and thrusting their head through the aperture, make attempts to bite in every direction. How long such a state continues, until death occurs spontaneously, I am not able to determine, as in the cases which came under my observation, the natural termination was not waited for.

4. *In Pigs.* During my long continuance in office, I met with but a solitary instance of rabies in the pig. This animal, a fattening boar, had been bitten, four days previously to my seeing it, by a mad dog, but it already raged with such violence, that I could only observe it through the crevices of the sty wherein it was inclosed. It gnashed its grinders, jumped up at the sides of the sty, at one instant, and threatened to burst through them, at the next, by the violence of its efforts. I directed it to be killed, and, together with the cleansings from the sty, to be buried at a great depth in the ground, which I was afterwards assured had been done. I subsequently discovered, however, that a butcher who had slain the animal with a hatchet, instead of allowing the carcass to be buried, had cut it up and exposed it for sale, and that, from its fatness, it had been readily disposed of. Notwithstanding the infamy of this act, aware that the possible evil consequences could no longer be averted, I too much dreaded the effects of the imagination in those who had partaken of the meat, to hazard a legal investigation on the subject. The disgraceful occurrence therefore slept in silence, but from that day to this—and it happened twenty-four years ago—I have not heard of any mischief arising from it.

5. *In Sheep.* In like manner, I have met with but one instance of rabies in sheep; in this one, however, five sheep, which were bit by a mad dog, all became affected at one and the same period, after the lapse of a few weeks. The symptoms, first noticed, were that the animals left off grazing and dispersed the flock by indiscriminate attempts to butt and to mount upon their fellows. They were all killed save one, which, being confined in a stall, kept quietly staring at the wall with a dejected look, as long as every thing around it was noiseless; but on hearing the slightest sound, the animal turned to the direction from whence it proceeded, and jumped up at the sides of the stall. Food and water were left untouched. Here my observations were cut short by the proprietor declining to await the natural termination. In a large flock, single cases of disease are usually overlooked until the symptoms become very conspicuous, and it appears more than probable that such was the fact in the present instance. The above details are therefore only to be received as a rough sketch.

The peasants would frequently hold fast the tongue of the rabid cattle with one hand, whilst they thrust the other deep into the creature's throat, in the endeavour to force nourishment into its stomach. That the naked hands and arms were on some of these occasions *not* entirely free from injury, I can scarcely doubt; and yet I never witnessed any evil effects to arise from the practice, although these people neglected to wash their hands subsequently. However, I knew a farmer to die of rabies, merely from having, with hands apparently uninjured, washed out the wound inflicted on a pig, by the bite of a rabid dog. On one occasion a slaughterer who had killed a rabid ox, in spite of my admonition, repeatedly thrust his naked arm into the animal's intestines without experiencing any evil therefrom, and cowherds, soi-disants veterinary surgeons, and the like, often commit similar acts with equal impunity.

I have frequently known the milk of rabid animals to be taken without detriment, and in two instances the flesh of rabid oxen which was clandestinely eaten, proved no less innoxious. It is nevertheless a fact that, at a period when many cattle perished of rabies, the instances of canine madness become unusually numerous; and it may be supposed that, however deeply the carcasses may be buried, dogs will still here and there succeed in disinterring them. Further experience is therefore requisite to decide on the effects of the saliva, blood, excrements, &c. in all domestic animals, with the exception of the dog and the cat. In conclusion, I have witnessed many instances where the bite of decidedly rabid animals has produced little or no effect on the human subject, although the remedies employed were merely such as were suggested by superstition. From this, I am led to infer, that with mankind a predisposition to hydrophobia very rarely exists.

*Hecker's Annalen.* 1st Band, Heft 4. 1836.

